# PROGNOSTIC PROFILES FOR DISCHARGE DESTINATION IDENTIFIED DURING INTENSIVE CARE UNIT ADMISSION: A DECISION TREE ANALYSIS

**DOI:** 10.2340/jrm.v58.44237

**Published:** 2026-05-20

**Authors:** Sho OTSUBO, Daisuke KAWAKAMI, Shota OKUNO, Kenta KAWAMITSU, Shumpei YOSHINO

**Affiliations:** 1 Department of Rehabilitation, Aso Iizuka Hospital, Iizuka, Fukuoka; 2 Department of Intensive Care Medicine, Aso Iizuka Hospital, Iizuka, Fukuoka, Japan

**Keywords:** decision trees, patient discharge, rehabilitation, prognosis, intensive care units

## Abstract

**Objective:**

This study aimed to develop a decision tree-based model to identify prognostic patient profiles associated with discharge destination during intensive care unit admission.

**Design:**

Historical cohort study using decision tree analysis.

**Subjects/Patients:**

300 critically ill patients selected from 857 intensive care unit admissions after applying exclusion criteria.

**Methods:**

The primary outcome was discharge destination (home vs non-home). Candidate variables included demographics, frailty, illness severity scores, delirium, ventilator duration, intensive care unit length of stay, and physical function on intensive care unit discharge. Terminal nodes were interpreted as prognostic profiles.

**Results:**

Decision tree analysis identified profiles based on Acute Physiology and Chronic Health Evaluation II score, intensive care unit length of stay, Functional Status Score for the Intensive Care Unit, and age. A profile with lower initial severity and prolonged stay was associated with non-home discharge (area under the curve 0.76). Conversely, a profile with higher initial severity, preserved functional mobility (Functional Status Score for the Intensive Care Unit ≥ 5), and age < 80 years was associated with home discharge (area under the curve 0.77).

**Conclusion:**

Combinations of illness severity, length of stay, functional status, and age-defined prognostic profiles were associated with discharge destination during intensive care unit admission.

## LAY ABSTRACT

Planning discharge for patients admitted to an intensive care unit (ICU) can be challenging. It is often difficult to predict whether a patient will be able to return home or require transfer to another facility after hospitalization. In this study, we used a decision tree method to identify patient “profiles” associated with discharge destination. These profiles were defined by combinations of illness severity, length of ICU stay, physical function on ICU discharge, and age. We found that patients with prolonged ICU stays despite lower illness severity were more likely to require non-home discharge. In contrast, patients with preserved minimal mobility and younger age were more likely to return home, even when illness severity was higher. These findings may help clinicians anticipate discharge needs and support early rehabilitation planning during ICU admission. Further studies are needed to confirm these results.

Patients requiring admission to an ICU are usually immobile for days to weeks owing to physical state, level of consciousness, and the need for intensive treatments leading to decline in cardiopulmonary function and skeletal muscle (1). It is known that a rapid loss of skeletal muscle mass occurs in more severely ill patients (2). Furthermore, it has been reported that approximately 30% of patients are in a frail state at the time of admission to the ICU (3) and that a high percentage of patients develop post-intensive care syndrome (4) after ICU admission, which often results in prolonged hospitalization, transfer to another hospital, or admission to a care facility (5, 6). On the other hand, there are patients who can be discharged home directly from the ICU without transferring to another hospital (7). If early and accurate predictions of home discharge or other destinations can be made while patients are still in the ICU, it enables early coordination for home discharge or admission to rehabilitation hospitals, long-term acute care hospitals, and other facilities. Such prognostic information may help clinicians anticipate discharge needs and support early multidisciplinary planning without restricting clinical decision-making. Therefore, this allows for the initiation of a targeted and intensive exercise programme for patients expected to be discharged home, ensuring that necessary rehabilitation is provided to those in need. These factors contribute to cost savings from reduced hospital stays (8–10) and, from a healthcare economic perspective, outcome prediction is both useful and essential, offering substantial benefits to patients and providers alike.

In recent years, the Functional Status Score for the ICU (FSS-ICU) (11, 12), age, pre-admission activities of daily living (ADL), and APACHE II score have been used to predict discharge home directly after admission via the ICU. Additionally, APACHE II score, length of hospital stay (13), ICU Mobility Scale score during ICU stay (14), and early mobilization (15, 16) have been reported to have a significant impact on patient outcomes. However, these reports typically rely on a single predictive factor, which may be insufficient for accurately predicting outcomes in ICU patients with diverse backgrounds. Additionally, given the large number of potential predictors, considering all of them individually is often impractical for making accurate predictions. We hypothesized that a combination model, which integrates multiple predictors in a user-friendly manner, could be beneficial in clinical practice.

Therefore, this study aimed to develop a predictive model of the optimal combination of factors that predict discharge destination during ICU admission.

## METHODS

### Study design and patient selection

A historical cohort study of a single ICU was conducted using medical records. Extraction and management of personal data were anonymized in accordance with the personal information protection law so that individual patients could not be identified.

**Fig. 1:**
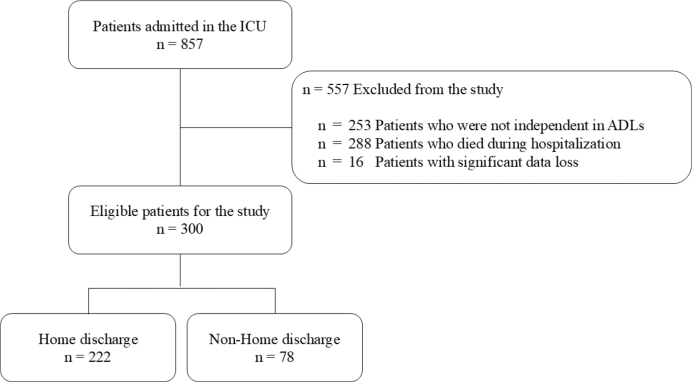
Flow diagram showing patients included in the study.

Eligible patients included all consecutive individuals aged 18 years or older who were admitted to the ICU (12 beds, semi-closed) between 1 September 2019, and 31 October 2023. Patients with non-independent pre-admission ADLs, death during hospitalization, and cases with significant data loss were excluded (Fig. 1). The investigated factors related to patient background included age, sex, body mass index, Clinical Frailty Scale (CFS), living alone status, presence of home modifications, need for step lifts at home, and caregiver certification status. Frailty was defined as a pre-admission CFS score of 4 or higher (17). Medical information included APACHE II score (18) on ICU admission, SOFA score, ICU length of stay, total hospital length of stay, presence of surgical intervention, ventilator duration (classified as ≥ 48 h or < 48 h, based on critical illness status according to previous studies [19]), number of physical therapy (PT) sessions, Charlson Comorbidity Index (CCI), presence of delirium in the ICU, FSS-ICU, and Medical Research Council Sum Score (MRCSS) on ICU discharge (20, 21). CCI was categorized as 2 points or less and 3 points or more (22). Delirium in the ICU was defined as an Intensive Care Delirium Screening Checklist (ICDSC) score of ≥ 4 points (23). Patient discharge destination was categorized as either home discharge or non-home discharge. Home discharge was defined as discharge to the patient’s original residence or a family member’s home. Non-home discharge included cases of admission to a nursing home or transfer to a rehabilitation hospital.

### Statistical analysis

Statistical analysis was conducted to compare the home discharge and non-home discharge groups. Continuous variables were expressed as mean (standard deviation) with or without normality; for 2-group comparisons, the χ^2^ test or Fisher’s exact probability test was used for categorical variables and the Mann–Whitney *U* test for continuous variables.

In this study, decision tree analysis was performed as a method to combine multiple factors to make predictions. Decision tree analysis is an analytical method in machine learning (24) that constructs a predictive model in the form of a tree diagram, identifying the optimal combination of various factors. Explanatory variables as combination factors were entered into the decision tree as factors affecting the possibility of discharge home or variables with *p* < 0.2 (25) in the comparison between the 2 groups and variables for clinical advantage. Continuous variables, including age, APACHE II score, ICU length of stay, SOFA score, FSS-ICU, MRCSS, BMI, CFS, and number of PT sessions, were entered into the decision tree model as continuous measures without pre-specified cut-off points. Certain variables were categorized prior to analysis based on clinically established thresholds reported in previous studies. These included ventilator duration ( < 48 h/ ≥ 48 h) (19), Charlson Comorbidity Index (≤2/ ≥ 3) (22), and delirium defined as an ICDSC score < 4 or ≥ 4 (23). All selected candidate variables were entered simultaneously into the decision tree model without prior stepwise selection. All thresholds presented in the tree were automatically determined by the algorithm based on optimal impurity reduction. Terminal nodes of the decision tree were interpreted as prognostic patient profiles. In this study, a “profile” refers to a subgroup of patients defined by a specific combination of predictors identified at a terminal node, each associated with a distinct probability of home discharge. The maximum depth of the tree was set to 3 and the minimum number of cases required prior to analysis was set at 20. All available data for each factor were submitted, including cases with missing data. Missing values were retained and handled internally by the decision tree algorithm without prior imputation. Receiver operating characteristic (ROC) curves were then created to evaluate the validity of the models obtained from the decision tree analysis, and the area under the curve (AUC), sensitivity, and specificity of each model were determined. A series of statistical analyses were performed using R software (R Foundation for Statistical Computing, Vienna, Austria, Version 4.2.2).

## RESULTS

**Fig. 2:**
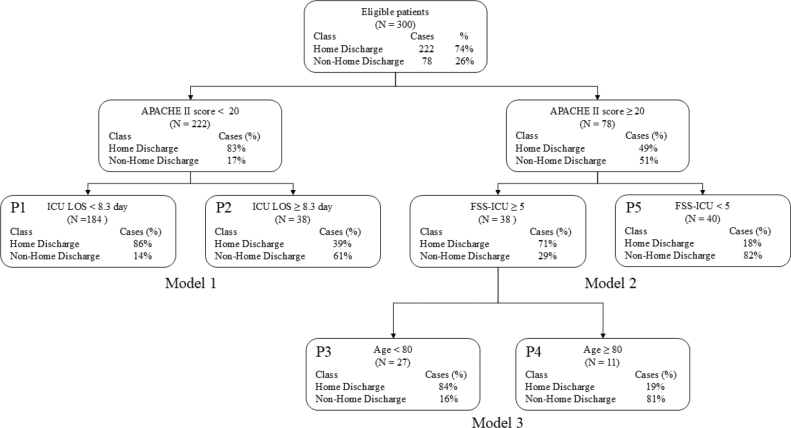
Decision tree model for the prediction of discharge destination. ICU LOS: ICU Length Of Stay. Model 1: APACHE II and ICU LOS. Model 2: APACHE II and FSS-ICU. Model 3: APACHE II, FSS-ICU and age, P: profile.

In this study, 857 patients were admitted to the ICU during the study period. Of these, 557 were excluded based on exclusion criteria: 253 had non-independent pre-admission ADLs, 288 died during hospitalization, and 16 had significant data loss. Consequently, 300 were included in the final analysis (see Fig. 1). The variables entered into the decision tree model and their data types are summarized in Table I. The subjects were classified into 2 groups, the home discharge group and the non-home discharge group, and the variables are presented in Table II; 222 (74%) were in the home discharge group and 78 (26%) were in the non-home discharge group. The mean age of the patients was 67.8 ± 15.2 years, with women comprising 33% of the cohort. The mean APACHE II score was 16.6 ± 6.4, and the mean SOFA score was 6.9 ± 3.5. Missing data were identified for certain variables, including 130 cases for the need for step lifts and 156 cases for home modifications. Comparison between the groups showed that significant differences were found in the following characteristics: age (66.4 ± 12.7 vs 71.8 ± 15.9; *p* = 0.006), days in ICU (3.9 ± 2.4 vs 6.6 ± 4.8; *p* < 0.001), APACHE II score (15.25.3 vs 20.4 ± 7.7; *p* < 0.001), SOFA score (6.4 ± 3.3 vs 8.4 ± 3.8; *p* < 0.001), duration of ventilator use ( ≥ 48 h: 17.6% vs 51.3%; *p* < 0.001), presence of home modifications (15.3% vs 20.5%; *p* = 0.012), surgery (Emergency 23% vs 34.6%, Elective 31.5% vs 10.3%, Non-surgery 45.5% vs 55.1%; *p* < 0.001), FSS-ICU (12.2 ± 8.5 vs 7.1 ± 6.9; *p* < 0.001), MRCSS (35.7 ± 25.6 vs 18.9 ± 23.1; *p* < 0.001), delirium (11.7% vs 24.4%; *p* = 0.012), CFS (3.1 ± 1.5 vs 3.5 ± 1.5; *p* = 0.058), need for step lifts (48.6% vs 38.5%; *p* = 0.088), and number of PT sessions (3.4 ± 2.8 vs 6.4 ± 6.3; *p* < 0.001). A decision tree analysis was conducted using these 13 factors, with a maximum depth of 3 branches (Fig. 2). The decision tree generated 5 terminal nodes which were defined as Profile 1 to Profile 5. These terminal nodes were interpreted as 5 prognostic profiles defined by distinct combinations of APACHE II score, ICU length of stay, FSS-ICU, and age. These profiles were subsequently interpreted as 3 broader prognostic groups corresponding to high, intermediate, and low probabilities of home discharge. The characteristics and outcome probabilities of each profile are summarized in Table III. In this decision tree analysis, 3 models were created. APACHE II score was selected in the first branch and split into 2 groups of less than 20 points and equal to or over 20 points. In Model 1, ICU length of stay was selected in the second branch for the group with an APACHE II score of less than 20 points. In Model 2, the group with an APACHE II score of 20 or higher, FSS-ICU was selected in the second branch. In addition, in Model 3, for patients with an FSS-ICU score of 5 or higher, age was selected as the third branch. ROC analysis was performed after logistic analysis for each prediction model (Table IV) and the AUC was compared. In Model 1 (APACHE II score; and ICU length of stay), the AUC was 0.76 with a sensitivity of 0.63 and a specificity of 0.82. In Model 2 (APACHE II score and FSS-ICU), the AUC was 0.75 with a sensitivity of 0.69 and a specificity of 0.69. In Model 3 (APACHE II score; FSS-ICU; and age), the AUC was 0.77, with a sensitivity of 0.85 and a specificity of 0.57.

**Table I:** Variables entered into the decision tree model

Variable	Data type in analysis	Coding/categorization
Age	Continuous	–
BMI	Continuous	–
ICU length of stay	Continuous	–
APACHE II score	Continuous	–
SOFA score	Continuous	–
CFS	Continuous	–
FSS-ICU	Continuous	–
MRCSS	Continuous	–
PT sessions	Continuous	–
Living alone	Binary categorical	0 = No, 1 = Yes
Sex	Binary categorical	Female, male
Duration of ventilation	Binary categorical	≥ 48h /< 48h
CCI	Binary categorical	CCI ≥ 3 / CCI ≤ 2
Need for step lifts	Binary categorical	Yes / No
Home modifications	Binary categorical	Yes / No
Surgery status	Binary categorical	Surgery / No surgery
Surgery urgency	Ordinal categorical	None / Elective / Emergency
Delirium (ICDSC)	Binary categorical	≥ 4 / < 4

BMI: body mass Index; APACHE II score: acute physiology and chronic health evaluation score; SOFA: Sequential Organ Failure Assessment score; CFS: Clinical Frailty Scale; FSS-ICU: functional status score for the ICU; MRCSS: Medical Research Council Sum Score; PT: physical therapy; CCI: Charlson Comorbidity Index; ICDSC: Intensive Care Delirium Screening Checklist.

**Table II:** Combination variables between home discharge and non-home discharge groups

Factor	Total (*n* = 300)	Home discharge (*n* = 222)	Non-home discharge (*n* = 78)	*p*-value
Age, years, mean (SD)	67.8 [15.2]	66.4 [14.7]	71.8 [15.9]	0.006
Female, n (%)	99 (33.0)	69 (31.1)	30 (38.5)	0.293
BMI (kg/m²), mean (SD)	23.5 [4.5]	23.6 [4.4]	23.4 [4.8]	0.752
CFS, mean (SD)	3.2 [1.5]	3.1 [1.5]	3.5 [1.5]	0.058
CCI, < 3, *n* (%)	77 (25.7)	59 (26.6)	18 (23.1)	0.647
Living alone, *n* (%)	68 (22.7)	48 (21.6)	20 (25.6)	0.567
Need for step lifts^a^, *n* (%)	*n* = 170	*n* = 134	*n* = 36	
	138 (46.0)	108 (48.6)	30 (38.5)	0.088
Home modifications^b^, *n* (%)	*n* = 144	*n* = 114	*n* = 30	
	50 (16.7)	34 (15.3)	16 (20.5)	0.012
APACHE II score, mean (SD)	16.6 [6.4]	15.2 [5.3]	20.4 [7.7]	< 0.001
SOFA score, mean (SD)	6.9 [3.5]	6.4 [3.3]	8.4 [3.8]	< 0.001
Surgery, *n* (%)				< 0.001
Emergency	78 (26.0)	51 (23.0)	27 (34.6)	
Elective	78 (26.0)	70 (31.5)	8 (10.3)	
Non-surgery	144 (48.0)	101 (45.5)	43 (55.1)	
Duration of ventilation, ≥ 48 h, *n* (%)	79 (26.3)	39 (17.6)	40 (51.3)	< 0.001
Delirium, ICDSC ≥ 4, *n* (%)	45 (15.0)	26 (11.7)	19 (24.4)	0.012
FSS-ICU, mean (SD)	10.9 [8.4]	12.2 [8.5]	7.1 [6.9]	< 0.001
MRCSS, mean (SD)	31.3 [26.0]	35.7 [25.6]	18.9 [23.1]	< 0.001
ICU length of stay, mean (SD)	4.6 [3.4]	3.9 [2.4]	6.6 [4.8]	< 0.001
PT sessions, mean (SD)	4.2 [4.3]	3.4 [2.8]	6.4 [6.3]	< 0.001

a Missing data: total 130 (43.3%): home discharge: 88 (39.6%) and non-home discharge: 42 (53.8%).

b Missing data: total 156 (52.0%): home discharge: 88 (39.6%) and non-home discharge: 48 (61.5%).

SD: standard deviation; BMI: body mass index; CFS: Clinical Frailty Scale; CCI: Charlson Comorbidity Index; APACHE II score: Acute Physiology And Chronic Health Evaluation score; SOFA: Sequential Organ Failure Assessment score; FSS-ICU: Functional Status Score for the ICU; MRCSS: Medical Research Council Sum Score; PT: physical therapy; ICDSC: Intensive Care Delirium Screening Checklist.

**Table III:** Prognostic patient profiles for discharge destination identified by decision tree analysis

Profile	APACHE II score	ICU LOS	FSS-ICU	Age	Probability of home discharge (%)	95% CI	Clinical interpretation
P 1	< 20	< 8.3 days	–	–	86%	80.9–90.3	Lower illness severity with short ICU stay
P 2	< 20	≥ 8.3 days	–	–	39%	19.8–64.3	Prolonged ICU stay despite lower illness severity
P 3	≥ 20	–	≥ 5	< 80	84%	70.3–92.8	Severe illness but preserved basic mobility and younger age
P 4	≥ 20	–	≥ 5	≥ 80	19%	5.7–51.0	Severe illness with advanced age
P 5	≥ 20	–	< 5	–	18%	6.9–31.8	Severe illness with marked functional limitation

APACHE II score: Acute Physiology And Chronic Health Evaluation score; ICU LOS: ICU Length Of Stay; FSS-ICU: Functional Status Score for the ICU; 95% CI: 95% confidence interval.

**Table IV:** Comparison of model performance measures (AUC, sensitivity, specificity)

Model	AUC	95% CI	Sensitivity	Specificity
Model 1^a^	0.76	0.70–0.83	0.63	0.82
Model 2^b^	0.75	0.69–0.82	0.69	0.69
Model 3^c^	0.77	0.71–0.83	0.85	0.57

a APACHE II score and ICU length of stay,

b APACHE II score and FSS-ICU,

c APACHE II score, FSS-ICU and age.

APACHE II score: acute physiology and chronic health evaluation; FSS-ICU: functional status score for the ICU.

## DISCUSSION

This study identified 3 key findings. First, APACHE II score, ICU length of stay, FSS-ICU, and age were found to be relevant factors in predicting discharge destination. Second, a combination of illness severity and ICU length of stay was related to non-home discharge. Third, a combination of illness severity, functional status, and age was related to home discharge.

First, in this study, APACHE II score, ICU length of stay, FSS-ICU, and age were identified as factors associated with predicting discharge destination. APACHE II score has been reported to be predictive of prognosis in ICU patients (26) and to be related to discharge home after discharge from the ICU (13), so in this study it was also one of the factors that predicted home discharge. With regard to ICU length of stay, longer lengths of stay are associated with increased in-hospital and long-term mortality (26, 27), as well as with decreased long-term health-related quality of life and physical function (27). Additionally, due to the nature of the ICU, the decrease in activity associated with admission to the ICU may cause a decline in musculoskeletal and cardiopulmonary function (1), which may well affect patient outcome, so the number of days in the ICU was extracted as a predictive factor. FSS-ICU evaluates the patient’s level of independence in basic movement skills, with a cutoff score of 19–23 points indicating eligibility for discharge home (11, 12). Previous studies have identified it as a predictor of discharge destinations, demonstrating advantages over other physical function assessment measures, such as minimal floor and ceiling effects and the absence of special tools, thereby enhancing its versatility (14, 28). Age has been reported as a poor prognostic factor (29) and a risk factor for cognitive decline (30). Additionally, ageing has been shown to influence functional prognosis (31) and significantly impact patient outcomes. Especially in Japan, the increasing age of patients is one of the most critical factors affecting outcomes. All 4 of these factors are characterized by assessments that are simple, versatile, and reproducible (32, 33). While these factors have been individually reported as prognostic indicators in ICU populations, the present decision tree analysis suggests that their combinations define clinically interpretable patient profiles with different probabilities of home discharge. In this study, a “profile” refers to a subgroup of patients defined by a specific combination of predictors identified at the terminal nodes of the decision tree, each associated with a distinct probability of home discharge. In other words, these factors should be interpreted as components within prognostic profiles rather than independent predictors.

Second, a profile characterized by lower illness severity but prolonged ICU stay was associated with a substantially reduced probability of home discharge. Although ICU length of stay tends to be shorter in patients with lower APACHE II scores (34), prolonged ICU stay in this subgroup may reflect accumulated clinical complexity not fully captured by baseline severity, including complications or delayed recovery. Previous studies have reported that lower APACHE II scores and shorter ICU stays are associated with favourable discharge outcomes (13, 27), and that prolonged ICU stays are linked to worse long-term functional outcomes (27, 31). Therefore, ICU length of stay in this context may function as an integrative marker of recovery trajectory rather than a purely temporal variable. This interpretation may explain why patients with relatively low illness severity but extended ICU stays showed a markedly reduced probability of home discharge.

Third, a profile characterized by higher illness severity but preserved minimal functional mobility and younger age was associated with a high probability of home discharge. Although greater illness severity is generally associated with poorer prognosis, preserved functional status at ICU discharge may mitigate the negative impact of severity. Previous studies have demonstrated that FSS-ICU is strongly associated with discharge destination (11, 12), and lower FSS-ICU scores have been linked to poor outcomes (28). A score of 5 or higher on the FSS-ICU typically indicates that the patient has some preserved functional abilities but is still limited. This suggests that the patient is expected to be able to disengage from the bed, even if only slightly and with assistance. Therefore, it is likely that the FSS-ICU scores were calculated as lower in the severely ill patients than in the previous study, because being able to move even a little is important for predicting discharge destinations in the severely ill. In addition, advanced age has been associated with increased mortality (35) and lower likelihood of direct home discharge (36), particularly among patients older than 80 years. While illness severity alone may provide reasonable prognostic discrimination, particularly among patients with lower APACHE II scores, the present findings suggest that severity scores alone may not fully capture discharge potential among patients with higher illness severity, where functional reserve and age appear to play important modifying roles. From the perspective of rehabilitation medicine, this profile-based framework may support early discharge planning and individualized rehabilitation strategies during ICU admission. In this study, the factors that make up the extracted prediction model were not limited to one factor as in previous studies, but were composed of multiple factors; the study population was not classified into specific departments or groups, but comprised all patients admitted to the ICU; and all factor assessments were simple and had high reliability and reproducibility (32, 33) and can be completed while the patient is in the ICU. By identifying patient groups with distinct prognostic trajectories, clinicians may better anticipate rehabilitation needs and coordinate discharge destinations. Importantly, this model should be interpreted as a decision-support tool rather than a deterministic predictor of individual outcomes, which will help in practice.

Several limitations of this study should be acknowledged. This was a single-centre study using historical data, which may limit generalizability. Although the decision tree generated multiple terminal nodes, the model effectively discriminated a limited number of prognostic profiles. Specifically, the model distinguished 3 broad prognostic groups corresponding to high, intermediate, and low probabilities of home discharge. Cut-off points such as ICU length of stay should therefore be interpreted probabilistically rather than as strict thresholds. Some potentially relevant factors were not included and there is a possibility that various factors that could not be assessed may be identified as important. In addition, missing data for certain variables may have influenced model stability. Finally, external validation was not performed, raising the possibility of overfitting. Future studies should validate these profiles in independent cohorts and examine whether incorporating additional functional or social variables improves prognostic resolution.

In conclusion, APACHE II score, ICU length of stay, FSS-ICU, and age were associated with discharge destination during ICU admission. The decision tree analysis suggests that combinations of these factors define prognostic profiles that may support early discharge planning. Further validation is warranted to confirm the generalizability and clinical utility of this model.
